# Lanthanum carbonate for the treatment of hyperphosphatemia in CKD 5D: multicenter, double blind, randomized, controlled trial in mainland China

**DOI:** 10.1186/1471-2369-14-29

**Published:** 2013-02-04

**Authors:** Jing Xu, Yi-Xiang Zhang, Xue-Qing Yu, Zhi-Hong Liu, Li-Ning Wang, Jiang-Hua Chen, Ya-Ping Fan, Zhao-Hui Ni, Mei Wang, Fa-Huan Yuan, Guo-Hua Ding, Xiang-Mei Chen, Ai-Ping Zhang, Chang-Lin Mei

**Affiliations:** 1 Department of Nephrology, Changzheng Hospital, Second Military Medical University, Shanghai, China; 2 Department of Nephrology, First Affiliated Hospital of Sun Yat-sen University, Guangzhou, Guangdong, China; 3 Department of Nephrology, Institute of Kidney Disease of the Chinese People's Liberation Army, Jingling Hospital, Nanjing University School of Medicine, Nanjing, China; 4 Department of Nephrology, First Affiliated Hospital of China Medical University, Shenyang, China; 5 Kidney Disease Center, First Affiliated Hospital, College of Medicine, Zhejiang University, Hangzhou, China; 6 Department of Nephrology, Affiliated Hospital of Nantong Medical College, Jiangsu, China; 7 Renal Division, Renji Hospital, Shanghai Jiaotong University School of Medicine, Shanghai, China; 8 Department of Nephrology, People's Hospital of Peking University, Beijing, China; 9 Institute of Nephrology of Chongqing and Department of Nephrology, Xinqiao Hospital, Third Military Medical University, Chongqing, China; 10 Department of Nephrology, Renmin Hospital of Wuhan University, Wuhan, China; 11 Chinese PLA Institute of Nephrology, Chinese PLA General Hospital and Military Medical Postgraduate College, Beijing, China; 12 Department of Nephrology, General Hospital of Jinan Military Command, Jinan, Shandong, China

**Keywords:** Lanthanum carbonate, Hyperphosphatemia, Chronic kidney disease 5D, Hemodialysis, Continuous ambulatory peritoneal dialysis (CAPD)

## Abstract

**Background:**

Serum phosphorus control is critical for chronic kidney disease (CKD) 5D patients. Currently, clinical profile for an oral phosphorus binder in the mainland Chinese population is not available.

**Objective:**

To establish the efficacy, safety, and tolerability of lanthanum carbonate in CKD 5D patients.

**Design:**

Multicenter, randomized, double blind, placebo-controlled study. A central randomization center used computer generated tables to allocate treatments.

**Setting:**

Twelve tertiary teaching hospitals and medical university affiliated hospitals in mainland China.

**Participants:**

Overall, 258 hemodialysis or continuous ambulatory peritoneal dialysis (CAPD) adult patients were enrolled.

**Intervention:**

After a 0–3-week washout period and a 4-week lanthanum carbonate dose-titration period, 230 patients were randomized 1:1 to receive lanthanum carbonate (1500 mg-3000 mg) or placebo for a further 4-week maintenance phase.

**Main outcome measures:**

Efficacy and safety of lanthanum carbonate to achieve and maintain target serum phosphorus concentrations were assessed.

**Results:**

In the titration phase, serum phosphorus concentrations of all patients decreased significantly. About three-fifths achieved target levels without significantly disturbing serum calcium levels. At the end of the maintenance period, the mean difference in serum phosphorus was significantly different between the lanthanum carbonate and placebo-treated groups (0.63±0.62 mmol/L vs. 0.15±0.52 mmol/L, P < 0.001). The drug-related adverse effects were mild and mostly gastrointestinal in nature.

**Conclusion:**

Lanthanum carbonate is an efficacious and well-tolerated oral phosphate binder with a mild AE profile in hemodialysis and CAPD patients. This agent may provide an alternative for the treatment of hyperphosphatemia in CKD 5D patients in mainland China.

**Trial registration:**

No. ChiCTR-TRC-10000817

## Background

Hyperphosphatemia is highly prevalent in chronic kidney disease (CKD) 5D patients undergoing hemodialysis and peritoneal dialysis [1]. Uncontrolled hyperphosphatemia contributes to the development of secondary hyperparathyroidism, renal osteodystrophy, vascular calcification, and a graded increase of all-cause mortality in dialysis patients [2-6]. There is also an association between increased phosphate levels, and mortality and cardiovascular mortality risk [7,8]. The K/DOQI 2003 guidelines recommend a target phosphatemia of 3.5–5.5 mg/dL (1.21–1.78 mmol/L) in dialysis patients [9]. Despite dietary restriction and adequate dialysis, 90% of dialysis patients still need oral phosphate binders to control their phosphate levels and thereby reduce mortality [1,10,11].

Aluminum hydroxide used to be the mainstay of phosphate-binding therapy but was largely abandoned due to its systemic toxicity. Calcium-based phosphate binders (carbonate or acetate) are the most commonly used phosphate binders in contemporary practice, although to date few randomized controlled trials (RCTs) have examined their effect on improving prognosis and decreasing mortality. Moreover, these phosphate binders may cause hypercalcemia in ≤50% of treated patients, leading to vascular calcification and higher death risk, especially when co-administered with vitamin D analogs [3,4,12,13]. Sevelamer hydrochloride/carbonate is the first synthetic non-calcium, non-aluminum phosphate binder without the tendency to promote hypercalcemia. However, recent meta-analyses failed to establish comparative superiority of sevelamer over calcium-based phosphate binders [14,15]. Moreover, patients often require large numbers of tablets to achieve phosphorus control.

The limitations of current treatments for hyperphosphatemia underscore the need for safe and efficacious calcium- and aluminum-free alternatives with low tablet load. Lanthanum carbonate (Fosrenol®) is a novel phosphate binder with similar therapeutic potency as aluminum hydroxide but a more favorable safety profile. Several RCTs have demonstrated lanthanum carbonate to be an efficacious and well tolerated agent for phosphorus control with low tablet burden in CKD 5D patients [16-20]. A double-blind RCT in 73 Chinese patients on hemodialysis in Taiwan showed similar results to previous US and European studies [17]. However, mainland China has more limited medical resources and must serve a larger population of CKD 5D patients across a broader territory than other major regions of China, including Taiwan, which makes comprehensive treatment of these patients more difficult and challenging [21]. Furthermore, there are currently no officially approved phosphorus binders in mainland China. Therefore, there is an urgent need to establish evidence-based research on oral phosphate-binders to guide serum phosphorus control of CKD 5D patients in mainland China.

To establish the clinical profile of lanthanum carbonate in the mainland Chinese population and identify any difference from other countries, a multicenter, randomized, double-blind study was conducted. The study assessed the efficacy, safety, and tolerability of lanthanum carbonate to achieve and maintain target serum phosphorus concentrations in CKD 5D patients on hemodialysis or continuous ambulatory peritoneal dialysis (CAPD). Changes in other important parameters such as serum calcium and serum parathyroid hormone (PTH) levels were also monitored.

## Methods

### Patients

CKD 5D patients aged 18–70 years receiving hemodialysis or CAPD for ≥3 months were eligible to participate. Patients fulfilling any of the following criteria were excluded: 1) hypercalcemia (serum calcium >10.4 mg/dL [>2.60 mmol/L]) or hypocalcemia (serum calcium <8.4 mg/dL [<2.10 mmol/L]); 2) severe hyperparathyroidism (PTH >1000 pg/mL [>105.3 pmol/L]); 3) previous gastrointestinal (GI) surgery or ongoing GI dysfunction including uncontrolled ulcer, inflammatory bowel diseases, or GI bleeding in the past 6 months; 4) serum transaminases or bilirubin >2.5 times the upper limit of normal (ULN); 5) severe heart failure (NYHA class III–IV); and 6) other exclusion criteria included HIV-positive status, known allergy to lanthanum, pregnant or lactating women, life-threatening malignancy, and exposure to other experimental drugs within 30 days before screening. All patients were judged by the investigator to be compliant with the study protocol. Treatment was carried out strictly in accordance with the trial protocol. Patients with poor compliance or who failed to take medicine according to the protocol were also excluded. One subject (SP315) did not follow doctor’s orders and took α-keto acid tablets (3 tablets per day, 50 mg calcium per tablet, stopped after 10 days medication) in the titration phase. Because this occurred in the titration period, and a small dose of calcium (150 mg/day) was taken, the subject was not excluded.

### Study design

This was a phase III, randomized, double blind, placebo-controlled, dose-titration trial in hemodialysis or CAPD patients conducted simultaneously at 12 hospitals in mainland China (Registration No. ChiCTR-TRC-10000817). The study comprised three phases: screening and a 0–3-week washout period; a 4-week dose titration period; and a 4-week double blind, placebo-controlled, randomized, maintenance phase.

### Phase 1: screening/washout

A complete medical history and physical examination were performed at the screening visit. Patients who had not taken any phosphorus binders for 1 week before screening could skip the washout period. Patients previously on phosphate binders (calcium, sevelamer), on the other hand, discontinued them and underwent washout over 0–3 weeks. Patients were put on a low-phosphorus diet (800–1000 mg/d). The calcium concentration of dialysis fluid and the mode of dialysis were kept constant. Phosphorus levels were monitored at each weekly visit. Patients whose serum phosphorus increased to >5.5 mg/dL (>1.78 mmol/L) were eligible to enter the dose-titration phase, while those whose phosphorus levels remained ≤5.5 mg/dL (≤1.78 mmol/L) at the end of week 3 of the washout period were withdrawn from the study.

### Phase 2: open-label dose titration

During the open-label dose-titration phase, all recruited patients received a starting daily dose of 1500 mg lanthanum carbonate and could be uptitrated to ≤3000 mg/d as necessary to achieve and maintain serum phosphorus ≤5.5 mg/dL (≤1.78 mmol/L, KDOQI 2003) over 4 weeks [9]. Patients on hemodialysis were titrated weekly, while those on CAPD were titrated every other week. A standardized dose-titration regimen based on serum phosphorus concentration was used. The dose of lanthanum carbonate was uptitrated one level (500 mg) in hemodialysis or two levels (1000 mg) in CAPD patients at each visit, if the serum phosphorus target level had not been achieved.

### Phase 3: double-blind randomization and maintenance treatment

At the end of the dose-titration phase, patients were randomized 1:1 to receive lanthanum carbonate or placebo for a further 4-week maintenance phase. Patients visited once every 2 weeks. No dose adjustments were given during this phase.

### Efficacy and safety assessments

The primary efficacy measure was the serum phosphorus level at the end of the maintenance phase compared with baseline (time of randomization). Secondary evaluation parameters involved serum phosphorus levels at each visit, proportion of patients with controlled serum phosphorous levels or response to the experimental drugs (defined as a decrease in the serum phosphorus level >25% from baseline) at the end of the maintenance phase, and intact PTH (iPTH) level at the end of the titration and maintenance stage.

Demographic characteristics such as sex, age, and medical history were collected at screening, whereas vital signs and concomitant medications were monitored throughout the study. Serum calcium levels were examined at each visit. Hematologic and biochemical parameters were measured at the end of each phase. Adverse events (AEs) and serious adverse events (SAEs) were recorded throughout the study.

### Statistical analysis

Assuming the mean difference of the serum phosphorus level compared to randomization between lanthanum carbonate and placebo to be −0.45 mmol/L, with a type I error of 2.5%, a power of 80%, and 20% loss to follow-up, it was calculated that about 120 patients per group were needed to detect the estimated differences, among whom 2/3 were hemodialysis patients, and 1/3 were peritoneal dialysis patients.

A blind data review meeting that involved statisticians, the data administrator, principal investigators, and the sponsor was conducted to divide the study population. According to the basic principles of intention-to-treat (ITT) analysis, all subjects who were randomized and had at least one record of drug administration and efficacy evaluation after randomization were included in the full analysis set (FAS) [22]. For the missing values of the main indicators (phosphate levels), the last observation carried forward (LOCF) estimation method was adopted when analyzing the FAS. All subjects who completed the treatment according to protocol or had no serious violations of the protocol were defined as the per-protocol set (PPS). For efficacy endpoints, FAS and PPS were analyzed statistically as the analysis set. Taking the analysis result of FAS as the main analysis result of the study and referring to the PPS analysis result, discussion and analysis were undertaken when inconsistencies occurred. For safety endpoints, the safety set (SS) was defined as a patient receiving at least one medication. The results were divided into titration and maintenance phases, and safety analysis was performed on the corresponding SS. For the primary efficacy analysis, serum phosphorus from randomization to each visit of maintenance phase was assessed by an analysis of covariance (ANCOVA) model, and the 95%CI was calculated. The percent of patients in each treatment group achieving target serum phosphorus was calculated and compared by the Cochran-Mantel-Haenszel test. The change in the serum phosphorus level at each visit over the titration period was examined. Continuous variables are expressed as means (± standard deviation) or medians (range) as appropriate, and they were compared by *t*-test or Wilcoxon rank-sum analysis. Categorical variables are expressed as numbers (percent) and were analyzed by the chi-squared test or Fisher’s exact test. Two-sided *P*-values < 0.05 were considered statistically significant. All statistical procedures were performed using SAS software (version 9.2, SAS Institute Inc, Cary, NC).

### Ethics

The study was implemented in compliance with the Declaration of Helsinki and approved by local ethics committees at each of the participating centers. Written, informed consent was obtained from all patients. The full name of the overall 12 ethical committees that granted approval for our study are as follows:

1. The ethics committee of Changzheng Hospital,

2. The ethics committee of First Affiliated Hospital of Sun Yat-sen University,

3. The ethics committee of Jingling Hospital,

4. The ethics committee of First Affiliated Hospital of China Medical University,

5. The ethics committee of First Affiliated Hospital of Zhejiang University,

6. The ethics committee of Affiliated Hospital of Nantong Medical College,

7. The ethics committee of Renji Hospital,

8. The ethics committee of People's Hospital of Peking University,

9. The ethics committee of Xinqiao Hospital,

10. The ethics committee of Renmin Hospital of Wuhan University,

11. The ethics committee of Chinese PLA General Hospital,

12. The ethics committee of General Hospital of Jinan Military Command.

## Results

### Patients

**Figure 1:**
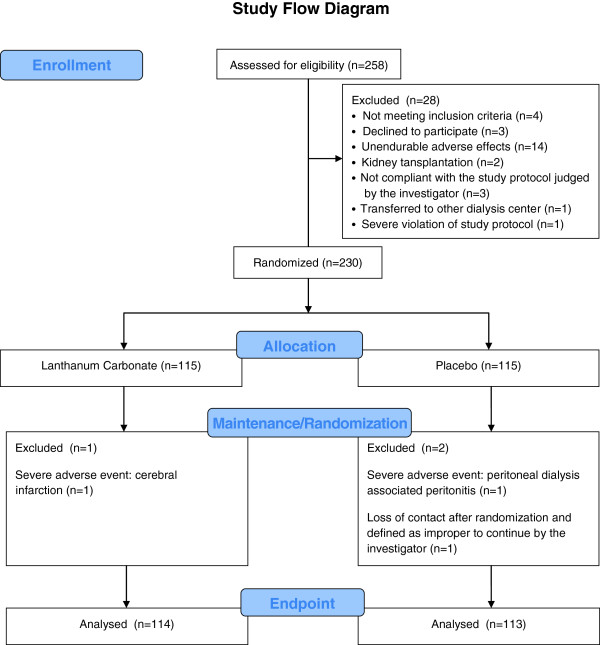
Study flow diagram.

A total of 258 consecutive patients entered the titration phase (Figure 1). During this period, 28 patients were withdrawn or excluded. The remaining 230 patients completed the titration phase and were randomized 1:1 into the maintenance phase. A further three patients were excluded at the blind data review meeting because none of them had validated efficacy evaluation after randomization. Among these three patients, two were in the control group. One had a cerebral infarction four days after randomization; the other patient could not be contacted by the investigator after randomization and was defined as inappropriate to continue. The remaining excluded patient was a peritoneal dialysis patient in the lanthanum carbonate group who developed peritonitis 10 days after randomization and stopped the medication. There was no significant difference in demographic features between lanthanum carbonate and placebo-treated patients (Table 1). Compliance with study treatment was similar in the two treatment groups (lanthanum carbonate, 92.9%; placebo, 94.6%; P = 0.59); for all patients, compliance was 93.7%.

**Table 1:** Demographics of all treated patients

**Items**	**Classification**	**Lanthanum carbonate(n=114)**	**Placebo(n=113)**	**P value**
Age (y)		47.6 (13.0)	48.4 (11.7)	0.761
Gender	Male	60 (52.6)	72 (63.7)	0.090
	Female	54 (47.4)	41 (36.3)	
Ethnicity	Han	112 (98.2)	111 (98.2)	1.000
	Other	2 (1.7)	2 (1.8)	
Height (cm)		165.7 (7.7)	165.9 (7.6)	0.674
Post-dialysis weight (kg)		60.9 (12.1)	61.9 (10.8)	0.342
Dialysis mode	Hemodialysis	82 (72.0)	82 (72.6)	0.915
	Peritoneal dialysis	32 (28.1)	31 (27.4)	
Hemodialysis frequency (per week)		2.9 (0.3)	2.8 (0.3)	0.553
Hemodialysis time (hr)		4.1 (0.3)	4.1 (0.3)	0.460
Total volume of peritoneal dialysate (ml)		7951.2 (1208.0)	7983.9 (1369.2)	0.839
Heart failure		4 (3.5)	2 (1.8)	0.683

### Serum phosphorus

**Figure 2:**
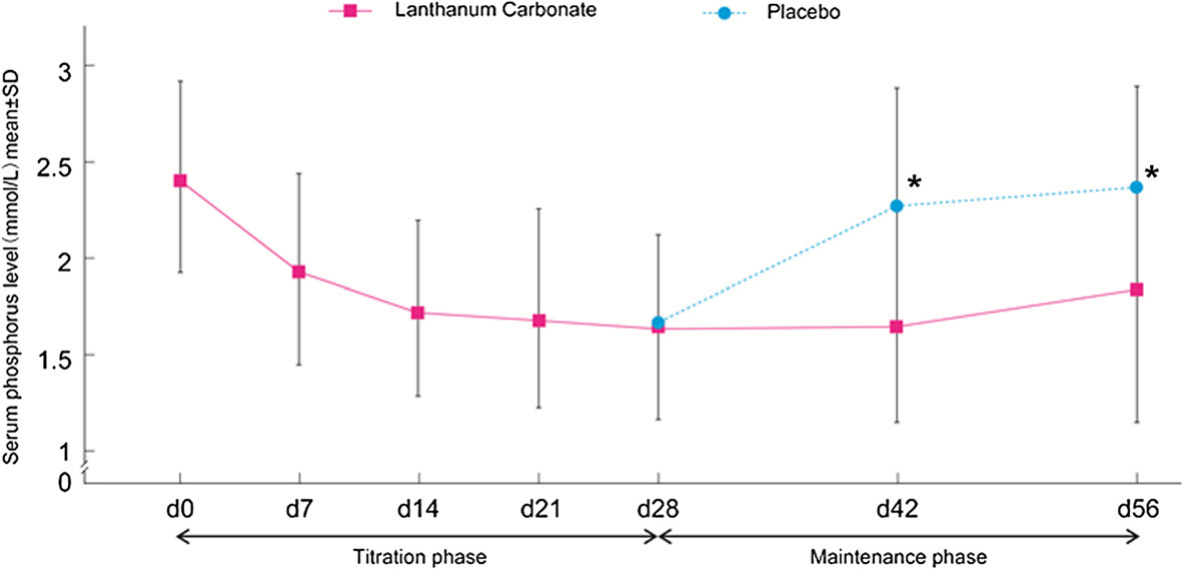
**Change in serum phosphorus concentrations at each visit over the entire study.** Data are expressed as means and standard deviations.

The mean serum phosphorus concentrations at each visit during the study are summarized in Figure 2 and Table 2. At the end of the washout period, the serum phosphorus concentration for all patients was 2.41 mmol/L. During dose titration with lanthanum carbonate, serum phosphorus concentration decreased; for patients assigned to lanthanum carbonate and placebo treatment groups this parameter was 1.64 and 1.71 mmol/L, respectively, at randomization (P = 0.24). During the maintenance phase, the mean serum phosphorus remained low for the lanthanum carbonate group but was substantially increased in the placebo group (Figure 2). Mean differences between the two groups were significant throughout randomized treatment (P < 0.001 at all time-points, Table 3). Compared with baseline (time of randomization), variations in the serum phosphorus level at the end of the maintenance phase were significantly lower in patients treated with lanthanum carbonate than in those on placebo: 0.15 mmol/L vs. 0.63 mmol/L (mean difference between groups of −0.48 [95% confidence interval (CI): -0.63, -0.33], P < 0.001).

**Table 2:** Mean serum phosphorus concentrations at each visit over the entire study

	**Total(n=227)**		
**Visiting time (d)**	**Lanthanum carbonate (n=114)**	**Placebo (n=113)**	**P value**
0	2.41 (0.50)	2.41 (0.50)	
7	1.90 (0.55)	1.93 (0.47)	
14	1.72 (0.41)	1.75 (0.51)	
21	1.70 (0.53)	1.74 (0.53)	
28	1.64 (0.46)	1.71 (0.49)	0.242
42	1.67 (0.51)	2.26 (0.61)	<0.001
56	1.79 (0.63)	2.34 (0.56)	<0.001

**Table 3:** Mean difference in serum phosphorus compared to baseline at each visit in the maintenance period

**Change in serum phosphorus from baseline**	**Lanthanum carbonate**	**Placebo**	***P *value**	**Mean(95%CI)(Lanthanum carbonate- Placebo)**
**FAS**				
Day 42 – Day 0				
No. of patients (Missing)	113(1)	113(0)		
Mean (SD)	0.04(0.52)	0.55(0.63)	< 0.001	−0.51(−0.67, -0.36)
Day 56 – Day 0				
No. of patients (Missing)	113(1)	113(0)		
Mean(SD)	0.15(0.52)	0.63(0.62)	< 0.001	−0.48(−0.63, -0.33)
**Hemodialysis subgroup**				
Day 42 – Day 0				
No. of patients (Missing)	81(1)	82(0)		
Mean(SD)	0.07(0.59)	0.53(0.71)	< 0.001	−0.46(−0.66, -0.25)
Day 56 – Day 0				
No. of patients (Missing)	81(1)	82(0)		
Mean(SD)	0.20(0.57)	0.62(0.68)	< 0.001	−0.42(−0.61, -0.22)
**Peritoneal dialysis subgroup**				
Day 42 – Day 0				
No. of patients (Missing)	32(0)	31(0)		
Mean(SD)	−0.06(0.24)	0.61(0.33)	0.000	−0.67(−0.82, -0.53)
Day 56 – Day 0				
No. of patients (Missing)	32(0)	31(0)		
Mean(SD)	0.01(0.32)	0.65(0.42)	0.000	−0.64(−0.83, -0.46)

After 4 weeks titration, 61.6% of patients had controlled serum phosphorus levels. At the end of the maintenance phase, compared with only 13.3% patients in the placebo group, 57.9% patients in the lanthanum carbonate group had serum phosphorus <1.78 mmol/L (P = 0.0001); more patients responded to the experimental drug (defined as a decrease of the serum phosphorus level >25% from baseline) in the lanthanum carbonate group than in the placebo group (56.1% vs. 12.4%; P = 0.0001). The primary efficacy measure and secondary evaluations all showed that lanthanum carbonate achieved better control of serum phosphorus than placebo.

In patients receiving lanthanum carbonate 1500 (40.3%, 46/114), 2000 (20.2%, 23/114), 2500 (26.3%, 30/114), or 3000 (13.2%, 15/114) mg and those on placebo, the proportion achieving the target serum phosphorus level (≤1.78 mmol/L) was 76.1%, 56.5%, 50.0%, 20.0%, and 13.3%, respectively.

### Serum calcium

At the end of the titration period, serum calcium decreased by 0.03 mmol/L versus the screening period (from 2.32 to 2.29 mmol/L; P = 0.014). Serum calcium in the lanthanum carbonate and placebo groups increased by 0.02 mmol/L and decreased by 0.02 mmol/L, respectively, at the end of the maintenance phase (mean difference between groups of 0.04 [95%CI: -0.03, 0.11], P = 0.035). However, the mean serum calcium level of the lanthanum carbonate-treated group (2.31 mmol/L) remained below the upper limit of normal (ULN) throughout the study.

### Ca × P product

Variation in the Ca × P product during the maintenance period from baseline (time of randomization) is shown in Table 4. The mean baseline Ca × P in the lanthanum carbonate-treated and placebo groups was 46.2 and 48.3 mg^2^/dL^2^, respectively (P = 0.17). During the second and fourth weeks of the maintenance phase, patients treated with lanthanum carbonate had Ca × P product levels of 47.3 and 50.9 mg^2^/dL^2^, respectively, both of which were below 55 mg^2^/dL^2^, as recommended by the KDOQI guideline [9]; whereas in the placebo group, this parameter increased significantly to 63.6 and 64.9 mg^2^/dL^2^, respectively, far exceeding the recommended level. At study end, the variation of Ca × P product was significantly different between the treatment groups (4.02 mmol/L vs. 17.46 mmol/L; mean difference between groups −13.44 [95%CI: -17.87,-9.00], P < 0.001).

**Table 4:** Mean difference in laboratory test results at the end of the maintenance period

**Change from Day 56 to baseline**	**Lanthanum carbonate**	**Placebo**	**P value**	**Mean(95% CI)(Lanthanum carbonate -Placebo)**
Calcium (mmol/L)				
No. of patients (Missing)	108(7)	110(5)		
Mean(SD)	0.02(0.32)	−0.02(0.19)	0.035	0.04(−0.03, 0.11)
Variation of Ca × P (mg^2^/dl^2^)				
No. of patients (Missing)	108(6)	109(4)		
Mean(SD)	4.02(15.02)	17.46(17.97)	< 0.001	−13.44(−17.87,-9.00)
iPTH (pg/ml)				
No. of patients (Missing)	109(5)	110(3)		
Mean(SD)	19.60(182.81)	53.63(136.88)	0.017	−34.03(−77.02,8.96)

iPTH, intact parathyroid hormone.

### Intact parathyroid hormone

At randomization, the difference in the mean iPTH level between the lanthanum carbonate and placebo-treated group was not significant (286.4 vs. 315.6 pg/mL; P = 0.48). Although the difference in the mean iPTH levels between the two groups remained not significant at the end of the maintenance phase, a significant difference in variation of the iPTH level from baseline was observed between the two groups: 19.60 mmol/L vs. 53.63 mmol/L (mean difference between groups −34.03 [95%CI: -77.02, 8.96], P = 0.017).

### Analysis of hemodialysis and peritoneal dialysis subgroups

The patients were divided into hemodialysis (n = 164) and CAPD (n = 63) subgroups. At the end of the maintenance period, the increment in serum phosphorus levels was significantly higher in placebo-treated patients than in lanthanum carbonate-treated individuals in both subgroups (P < 0.01 and = 0.0001, respectively; Table 3), similar to the results noted in the whole population.

### Safety evaluation

Over the titration period, 157 AEs were reported by 90 of 258 (34.9%) patients enrolled in the study; of these, 101 AEs reported by 40 patients (34.9%) were considered related to experimental drugs. No SAE or death was reported. Treatment-emergent AEs for the SS population during the titration phase are summarized in Table 5. AEs reported with the highest incidence were GI in nature, with nausea (15.5%) and vomiting (10.8%) the most common, accounting for 78.2% of overall events. Other common GI reactions were abdominal distension (3.1%), upper abdominal discomfort (1.9%), constipation (1.5%), abdominal pain (1.2%), and diarrhea (1.2%). The incidences of AEs other than the GI system were all <2%.

**Table 5:** Summary of drug-related adverse events during the titration period (no. cases >1)

**Symptoms**	**Cases**	**Number**	**Incidence(n=258)**
Nausea	48	40	15.50%
Vomiting	31	28	10.85%
Abdominal distention	7	7	2.71%
Epigastric discomfort	4	4	1.55%
Constipation	3	3	1.16%
Anorexia	4	4	1.55%
Abdominal pain	2	2	0.78%
**Total**	**101**	**90**	**34.88%**

The incidence of AEs was lower during the maintenance phase than the titration phase. There were 34 AEs in 19 of 115 patients (16.5%) in the lanthanum carbonate-treated group and 11 AEs in 10 of 115 patients (8.7%) in the placebo-treated group, with incidences of treatment-related AEs of 8.7% and 2.6%, respectively. Although the incidence of AEs was higher in the lanthanum carbonate-treated group than in the placebo-treated group, the difference was not significant. Treatment-emergent AEs for the SS population after randomization are shown in Table 6. Commonly reported AEs were nausea (6.9%), vomiting (6.1%), and hypocalcemia (1.7%). Apart from these, each AE reported by the two groups appeared in only one case.

**Table 6:** Summary of drug-related adverse events during the maintenance period

	**Lanthanum carbonate(n=115)**			**Placebo(n=115)**		
**Symptoms**	**Cases**	**Number**	**Incidence**	**Cases**	**Number**	**Incidence**
Nausea	12	8	6.96%		0	
Vomiting	11	7	6.09%		0	
Constipation		0		1	1	0.87%
Anorexia	1	1	0.87%		0	
Hypocalcemia		0		1	1	0.87%
Aggravated itching		0		1	1	0.87%
**Total**	**24**	**16**	**13.91%**	**3**	**3**	**2.61%**

Three SAEs were reported throughout the entire course of the study, all within the maintenance period. One case occurred in the lanthanum carbonate-treated group and two cases in the placebo-treated group; they were peritoneal dialysis-related peritonitis, cerebral infarction, and right cervical fibroma; none was considered related to experimental drugs. In total, 18 patients withdrew from the study because of unendurable AEs or SAEs during treatment phases. Fourteen patients withdrew within the titration phase and four within the maintenance phase. No deaths were reported during the overall study period.

## Discussion

This 8-week, multicenter, double-blind RCT conducted in mainland China demonstrated that lanthanum carbonate is efficacious and well tolerated for the treatment of hyperphosphatemia in hemodialysis and CAPD patients.

Although calcium-based phosphate binders, either calcium carbonate or calcium acetate, and aluminum hydroxide have been widely used to treat hyperphosphatemia in CKD patients, none of these agents has yet been registered and approved by the mainland Chinese State Food And Drug Administration (SFDA). Compared with more developed areas of China, such as Taiwan and Hong Kong, there are limited medical resources in mainland China, and these must serve a large population across a broad territory [21]. In 2002, nearly 1550 per million people (pmp) in Taiwan received regular dialysis treatment; this prevalence was even higher than that in the USA. In the same year, 550 pmp in Hong Kong and only 30 pmp in mainland China received such treatment [23]. Thus, CKD patients in mainland China urgently need to have available a validated and standardized oral phosphate binder to tackle phosphate retention [24]. To date, no RCT study from mainland China has investigated the efficacy and tolerability of oral phosphate binders. The current phase III study might contribute to lanthanum carbonate becoming the first certified oral phosphate-binding agent available for both hemodialysis and CAPD patients in mainland China.

The present study showed that serum phosphorus concentrations of all patients decreased gradually during the 4-week titration phase when they were treated by lanthanum carbonate. About three-fifths of patients treated with lanthanum carbonate achieved target serum phosphorus level. The primary efficacy measure and secondary evaluations all showed that lanthanum carbonate achieved better control of serum phosphorus than placebo (mean difference −0.48 mmol/L, median lanthanum carbonate dosage 2000 mg (data not shown)), without significantly disturbing serum calcium levels. Indeed, the level of serum calcium in the lanthanum carbonate group decreased by 0.03 mmol/L at the end of the titration phase compared with the screening period. The findings were not different between the hemodialysis and peritoneal dialysis subgroups. However, the change in the serum phosphorus concentration from baseline was similar to the level predicted at study design. Joy et al. [16] reported a mean difference of −0.6 mmol/L compared to placebo with a similar median dosage of about 2300 mg of lanthanum carbonate. In 2005, a study from Taiwan showed that lanthanum carbonate could achieve a −0.7 mmol/L mean difference compared to placebo with a much lower dosage of 750–1500 mg/d [17]. However, the present study sample was almost double and triple, respectively, of Joy’s study and Chiang’s study [16,17]. The efficacy of lanthanum carbonate should be further tested in future, long-term, RCT studies.

In patients receiving lanthanum carbonate 1500, 2000, 2500, and 3000 mg, target serum phosphorus (≤1.78 mmol/L) was achieved in 76.1%, 56.5%, 50.0%, and 20.0%, respectively, compared with 13.3% on placebo. These data suggest that lanthanum carbonate possesses high phosphorus-binding efficacy with low tablet burden. A single-dose balance study in healthy volunteers by Martin et al. [25] supports our results in that 1000 mg of lanthanum carbonate could decrease serum phosphate by 45% compared with a 21% decrease with 2400 mg of sevelamer carbonate. Daugirdas et al. [26] further calculated the phosphate binder equivalent doses. In their reports, 500 mg lanthanum carbonate was equivalent to 750 mg calcium carbonate, 667 mg calcium acetate, and 800 mg sevelamer carbonate, the phosphorus-binding capacity of which was equal to aluminum carbonate and not affected by the intestinal pH level [26,27]. Patients were put on a low-phosphorus diet (800–1000 mg/d) in the study. We also designed a dietary diary to monitor the dietary phosphorus intake of each patient (data not shown). However, the hospitals were understaffed and we did not have a specialized nutritionist to collect and calculate the data; so the higher doses prescribed might be partly due to higher dietary intakes, which could cause bias to the study. More emphasis should be given to this in future studies.

The Ca × P product of lanthanum carbonate-treated patients was below the level (55 mg^2^/dL^2^) recommended by the KDOQI guideline [9] at all visits both in the titration and maintenance phases, whereas that of placebo-treated individuals far exceeded it during the maintenance period. At study endpoint, the Ca × P product of the placebo group was 14.0 mg^2^/dL^2^ higher compared with the lanthanum carbonate group; the mean difference from baseline differed significantly (P < 0.001). The change in the iPTH level was also monitored throughout the study. The variation in the iPTH level from the time of randomization (19.60 vs. 53.63 pg/mL) between the two groups was significantly different (P = 0.017). These results support previous reports. Currently, bone biopsy cannot be performed clinically in mainland China. Although the present study could not demonstrate a beneficial effect of lanthanum carbonate on renal-associated osteopathy, the results indicate that lanthanum carbonate helped maintain the balance among Ca, P, and PTH.

Besides the good efficacy of lanthanum carbonate, the safety evaluation showed that lanthanum carbonate was generally safe and well-tolerated in CKD 5D patients. No deaths and few SAEs were reported throughout the study. The most frequently reported AEs were GI in nature, and they improved as patients moved from the titration to the maintenance phase. A similar pattern was observed by other researchers [16-20,28,29]. In all, 5.4% and 0.9% of patients withdrew during the titration and maintenance phases because of side effects of the experimental drugs. The incidence of AEs was not significantly different between lanthanum carbonate and placebo during the maintenance phase. The compliance of the present patients to the experimental drugs was quite good. Compared with previous double-blind reports, lanthanum carbonate was better tolerated in the present study. The present withdrawal rate during the titration and maintenance phases was the lowest, at only 10.8% and 6.2%, respectively (Additional file 1: Table S1) [16-20,30,31]. This could be partly due to the strong desire of CKD 5D patients in mainland China to have better treatment despite some mild discomfort taking the experimental drugs.

There are some limitations of the present study. Up to the time the study was launched, no patented calcium or non-calcium phosphate binder had been approved in mainland China. Although calcium carbonate was prescribed by nephrologists in some tertiary Chinese hospitals, it had not received certification from the government, and it could not be approved by the ethics committee. Thus, a calcium or non-calcium containing phosphate binder could not be used as a control group treatment.

Plasma lanthanum concentrations could not be monitored during the present study. Although lanthanum has been found to deposit in patients’ bone through biopsy, no toxicity similar to that of aluminum has been reported so far [32,33]. Several follow-up studies even showed positive effects of lanthanum carbonate on bone mineralization. Although follow-up studies, the longest of which was ≤6 years, detected no toxicity effect of lanthanum carbonate deposition on specific human organs, evaluation of the long-term tolerability and safety of lanthanum carbonate in the Chinese populations needs further study [34,35].

The treatment duration of the present study was only 8 weeks. No protective effect of lanthanum carbonate on patients’ survival or organ calcification was identified. Lanthanum carbonate has been shown to attenuate vascular calcification both in dialysis patients and in animal models [36,37]. Further studies should be conducted to evaluate the effect of lanthanum carbonate on the prognosis of CKD 5D patients in mainland China.

## Conclusions

In conclusion, the results of the present study suggest that lanthanum carbonate is an efficacious and well-tolerated oral phosphate binder with a mild AE profile in both hemodialysis and CAPD patients. This agent may provide an alternative for the treatment of hyperphosphatemia in CKD 5D patients in mainland China.

## Abbreviations

AEs: Adverse events; CAPD: Continuous ambulatory peritoneal dialysis; CI: Confidence interval; CKD: Chronic kidney disease; FAS: Full analysis set; GI: Gastrointestinal; ITT: Intention-to-treat; LOCF: Last observation carried forward; pmp: Per million people; PPS: Per-protocol set; PTH: Parathyroid hormone; RCT: Randomized controlled trial; SAEs: Serious adverse events; SFDA: State food and drug administration; SS: Safety set; ULN: Upper limit of normal.

## Competing interests

This study was supported by Fresenius-Kabi (China) Co., Ltd. None of the authors work, or hold any shares, in the company.

## Authors’ contribution

XJ helped with patient recruitment and implementation of the study, as well as conducting the literature review and preparation of the manuscript. ZYX helped with implementation of the whole study, supervised the field activities, and designed the study’s analytic strategy. YXQ, LZH, WLN, CJH, FYP, NZH, WM, YFH, DGH, CXM and ZAP were Chiefs in 11 of the centers, and took part in the implementation of the study. MCL was the Principle Investigator of this multicenter RCT; he also designed the study and directed its implementation, including quality assurance and control. All authors read and approved the final manuscript.

## Pre-publication history

The pre-publication history for this paper can be accessed here:

http://www.biomedcentral.com/1471-2369/14/29/prepub

## Supplementary Material

Additional file 1: Table S1 Summary of double-blind RCT studies on lanthanum carbonate. Click here for file
