# Supplementary material for: Lanthanum carbonate for the treatment of hyperphosphatemia in CKD 5D: multicenter, double blind, randomized, controlled trial in mainland China
Source: BMC Nephrol. 2013 Feb 4;14:29. doi: 10.1186/1471-2369-14-29 (PMC3570485; doi:10.1186/1471-2369-14-29)
Supplement: Additional file 1: Table S1 — Summary of double-blind RCT studies on lanthanum carbonate. [file 1471-2369-14-29-S1.docx]

Supplemental Table. Summary of double-blind RCT studies on lanthanum carbonate.

|  | Country | Study Type | Ratio of randomization | Time of Study | Control | Enrolled CKD Patients | No | Withdrew (%) | Compliant |
| --- | --- | --- | --- | --- | --- | --- | --- | --- | --- |
| Mei et al, 2010 | Mainland China | Multicenter double-blind RCT | 1:1 | 11 weeks | Placebo | Hemodialysis and CAPD | 258 | 13.6% | 92.9% |
| Chiang et al, 2005 [17] | Taiwan, China | Double-blind RCT | 1:1 | 11 weeks | Placebo | Hemodialysis | 73 | 42.5% | 95% |
| Joy et al, 2003 [16] | USA | Multicenter double-blind RCT | 1:1 | 13 weeks | Placebo | Hemodialysis | 126 | 34.9% | 86% |
| Sprague et al, 2009 [32] | USA | Multicenter double-blind RCT | 2:1 | 12 weeks | Placebo | CKD stage 3 and 4 | 121 | 41.3% | > 80% |
| Finn et al, 2004 [33] | USA | Single center double- blind RCT | 1:1:1:1:1 | 11 weeks | Placebo | Hemodialysis | 145 | 37.2% | NA |
| Shigematsu et al, 2008 [19] | Japan | Multicenter double-blind RCT | 1:1 | 10 weeks | Calcium carbonate | Hemodialysis | 312 | NA | NA |
| Al-Baaj et al, 2005 [20] | UK | Multicenter double-blind RCT | 1:1 | 10 weeks | Placebo | Hemodialysis and CAPD | 59 | 42.4% | 94% |
| Shigematsu et al, 2008 [18] | Japan | Multicenter double-blind RCT | 1:1:1:1:1 | 9 weeks | Placebo | Hemodialysis | 156 | NA | NA |
